# Cost-Effectiveness of Collaborative Care for Depression in UK Primary Care: Economic Evaluation of a Randomised Controlled Trial (CADET)

**DOI:** 10.1371/journal.pone.0104225

**Published:** 2014-08-14

**Authors:** Colin Green, David A. Richards, Jacqueline J. Hill, Linda Gask, Karina Lovell, Carolyn Chew-Graham, Peter Bower, John Cape, Stephen Pilling, Ricardo Araya, David Kessler, J. Martin Bland, Simon Gilbody, Glyn Lewis, Chris Manning, Adwoa Hughes-Morley, Michael Barkham

**Affiliations:** 1 University of Exeter Medical School, University of Exeter, Exeter, United Kingdom; 2 Mood Disorders Centre, University of Exeter, Exeter, United Kingdom; 3 Centre for Primary Care at the Institute of Population Health, University of Manchester, Manchester, United Kingdom; 4 School of Nursing, Midwifery, and Social Work, University of Manchester, Manchester, United Kingdom; 5 Primary Care and Health Sciences, Keele University, Keele, United Kingdom; 6 Research Department of Clinical, Educational, and Health Psychology, University College London, London, United Kingdom; 7 School of Social and Community Medicine, University of Bristol, Bristol, United Kingdom; 8 Department of Health Sciences, University of York, York, United Kingdom; 9 Department of Health Sciences at Hull York Medical School, University of York, York, United Kingdom; 10 Mental Health Sciences Unit, University College London, London, United Kingdom; 11 Upstream Healthcare, Teddington, United Kingdom; 12 Department of Psychology, University of Sheffield, Sheffield, United Kingdom; Heinrich-Heine University, Faculty of Medicine, Germany

## Abstract

**Background:**

Collaborative care is an effective treatment for the management of depression but evidence on its cost-effectiveness in the UK is lacking.

**Aims:**

To assess the cost-effectiveness of collaborative care in a UK primary care setting.

**Methods:**

An economic evaluation alongside a multi-centre cluster randomised controlled trial comparing collaborative care with usual primary care for adults with depression (n = 581). Costs, quality-adjusted life-years (QALYs), and incremental cost-effectiveness ratios (ICER) were calculated over a 12-month follow-up, from the perspective of the UK National Health Service and Personal Social Services (i.e. Third Party Payer). Sensitivity analyses are reported, and uncertainty is presented using the cost-effectiveness acceptability curve (CEAC) and the cost-effectiveness plane.

**Results:**

The collaborative care intervention had a mean cost of £272.50 per participant. Health and social care service use, excluding collaborative care, indicated a similar profile of resource use between collaborative care and usual care participants. Collaborative care offered a mean incremental gain of 0.02 (95% CI: –0.02, 0.06) quality-adjusted life-years over 12 months, at a mean incremental cost of £270.72 (95% CI: –202.98, 886.04), and resulted in an estimated mean cost per QALY of £14,248. Where costs associated with informal care are considered in sensitivity analyses collaborative care is expected to be less costly and more effective, thereby dominating treatment as usual.

**Conclusion:**

Collaborative care offers health gains at a relatively low cost, and is cost-effective compared with usual care against a decision-maker willingness to pay threshold of £20,000 per QALY gained. Results here support the commissioning of collaborative care in a UK primary care setting.

## Introduction

Depression is a long term and disabling condition with well documented negative impacts on health status and on health care resources [1], [2]. In most cases the responsibility for treatment of people with depression falls on primary care [3], but the organisation of care in this setting can face many challenges. These include barriers between generalist and specialist mental health professionals, poor patient adherence to pharmacological treatment [4], and a shortage of specialists to provide psychological therapies [5]. Against this backdrop there is a growing role for organisational interventions to support the management of depression, and collaborative care has been shown to be an effective intervention that supports the organisation of health care for depression [6]. Collaborative care is a complex intervention, developed in the United States, incorporating a multi-professional approach to patient care; a structured management plan; scheduled patient follow-ups; and enhanced inter-professional communication [7]. In practice this is achieved by the introduction of a care manager into primary care, responsible for delivering care to patients with depression under supervision from a specialist, and for liaising between primary care clinicians and mental health specialists. Systematic reviews, of studies mostly conducted in the United States, demonstrate that collaborative care improves depression outcomes [6], [8], and a recent randomised control trial, the CADET Trial of collaborative care for depression in UK primary care, has shown it to be effective in the UK healthcare system [9]. Evidence indicates that collaborative care is cost-effective in the setting of the United States healthcare system [10], [11], however, evidence on the cost effectiveness of collaborative care for depression in the UK has been lacking until now [10], [11]. We report here the results of an economic evaluation of collaborative care versus usual care carried out in the UK alongside the CADET trial.

## Methods

### Study/Trial design

The aim of the CADET trial (Trial Registration Number: ISRCTN32829227) was to determine the effectiveness and cost-effectiveness of collaborative care when added to usual care compared to usual care alone in the management of patients with moderate to severe depression in UK primary care. The design of this multi-centre, two group cluster randomised controlled trial, and methods for the economic evaluation, are described in detail in the published protocol [12]. In brief, we recruited participants from electronic case records of general practices in three UK sites: Bristol, London and Greater Manchester. Recruitment included patients newly identified as depressed, with or without one or more previous depressive episodes, and those with an existing diagnosis of depression which was not responding to primary care management. Eligible participants were people aged 18+ who met ICD-10 criteria for a depressive episode on the revised Clinical Interview Schedule (CIS-R) [13]. Ethical approval for the trial on which this economic evaluation is based was given by the UK NHS Health Research Authority, NRES Committee South West (NRES/07/H1208/60). All participants gave written informed consent before taking part in the study. No minors/children were enrolled in the study. Written consent forms were co-signed by participant and the person taking consent, and were securely stored in both original form and electronically. The ethics approval included approval of both the procedure and the forms and documentation used for consent. We excluded those at high risk of suicide, psychosis, bi-polar disorder, those whose depression was associated with bereavement, people whose primary presenting problem was alcohol or drug abuse, and those receiving psychological treatment for their depression via mental health services. We randomly allocated practices into collaborative care or usual care, using a minimisation approach, to minimise imbalance between treatment groups, using site (Bristol, London. Manchester), Index of Multiple Deprivation (IMD) rank [14], number of GPs, and practice size.

### Interventions

Participants allocated to the control condition, treatment as usual (TAU), received care from their general practitioner according to usual clinical practice for these patients, including treatment by antidepressants and referral for other treatments, including UK Improving Access to Psychological Therapies (IAPT) services.

The collaborative care intervention consisted of usual care from the GP, and additionally care managers were expected to provide between 6–12 contacts with participants over a period of 14 weeks: with these expected to comprise 30–40 minutes for one initial face to face appointment followed by 15–20 minute telephone contacts thereafter. Contacts included: education about depression; medication management; behavioural activation [15]; and relapse prevention instructions [16]. Care managers provided GPs with advice on medication and regular updates on patient progress including medication adherence.

Care managers had existing UK mental health qualifications (psychological wellbeing practitioners, counsellors or nurses) and were then trained specifically to deliver the intervention within the collaborative care framework. They received weekly supervision from a specialist mental health professional, a clinical psychologist, psychiatrist, an academic GP with special interest in mental health or a senior nurse psychotherapist. Individual patients were discussed in supervision at least monthly, facilitated through a bespoke computerised patient management system (PC-MIS-http://www.pc-mis.co.uk).

### Outcome measures

Clinical effectiveness was assessed using depression severity as the primary outcome, measured by the Patient Health Questionnaire-9 (PHQ-9) [17]. The PHQ-9 is a nine-item questionnaire, which records the core symptoms of depression, with scores ranging from 0 to 27 where higher scores indicate a greater severity of depression. Secondary outcomes were quality of life using the SF-36 [18], worry and anxiety using the GAD-7 [19], patient satisfaction using the CSQ8 [20], health care service use using a patient questionnaire, and health state values (health-related quality-of-life) using the EQ-5D 3L [21], [22]. All measures were collected at baseline, four months and 12 months post randomisation (except patient satisfaction, measured at four months only).

### Cost-effectiveness analyses

In our economic evaluation we adopted the perspective of the UK NHS and Personal Social Services (Third Party Payer perspective), with a broader participant and carer perspective considered in sensitivity analyses. Our primary economic endpoint was the cost per quality-adjusted life-year at 12-month follow-up. To assess cost-effectiveness we estimated the additional cost for delivery of the collaborative care intervention, the costs associated with health and social care service use, and estimated quality-adjusted life-years (QALYs). QALYs are a commonly used summary measure of health-related quality-of-life, taking account of both quality and quantity of life, and are increasingly used to compare the cost-effectiveness of interventions across a broad range of health and related contexts [23]. We estimated QALYs over the 12-month trial follow-up, using the EQ-5D trial data, applying UK tariffs obtained from a UK general population survey to value the EQ-5D health states [24]. In addition, to inform sensitivity analyses, we used trial data from the SF-36 to estimate QALYs using the SF-6D, which presents tariffs obtained from a UK general population survey to value health states [25]. We derived QALY estimates using data from baseline, 4-month and 12-month assessments, applying the area-under-the-curve approach, a recognised approach for assessing repeated measures data [26], as recommended by Brazier and colleagues [23].

We collected resource use associated with delivery of the collaborative care intervention within the trial, comprising care manager contact time, and supervision of care managers by specialists. We collected other health and social care resource use by participants over the 12-month follow-up and data on informal care from friends/relatives and patient costs using self-report, interviewer administered, questionnaires (at 4 and 12-months; covering the prior 4-month and prior 8-month time periods respectively). We collected this same data at the baseline assessment, with participants reporting resource use over the 6-month period prior to baseline assessment.

We combined data on resource use with published unit costs to estimate mean cost per participant by using healthcare resource values from unit costs in nationally available data sources, adjusted for inflation where necessary, in British pounds sterling (£) at 2011 costs, see Table 1. In estimates of the intervention cost for collaborative care, we based care manager costs on costs for UK NHS Agenda for Change (AfC) band 5 staff costs, using a unit cost of £65 per hour for patient contact time [27], equivalent to Mental Health Nurse. This unit cost includes all staff cost components, and an allowance of contact time to non-contact time of 1∶0.89 (see [27], Table 10.2). We based supervision costs on a unit cost of £135 per hour for Clinical Supervisors, based on full costs for specialist mental health professionals at NHS AfC band 8a ([27], Table 9.5).

**Table 1:** Unit costs for different types of health and social care resource items.

Resource item	Unitcosts*	Source ofcost data	Basis of estimate
GP (at surgery/practice)	£36.00	PSSRU 2011	GP appt/surgery; based on costing at 11.7 mins
GP (at home)	£121.00	PSSRU 2011	
Practice Nurse (surgery)	£15.00	PSSRU 2011	15.5 min/hourly patient-related cost
Practice Nurse (home)	£30.00	PSSRU 2011	25 min/hourly home visiting cost
Walk-in-centre (appt)	£41.00	PSSRU 2011	Walk-in-service (not admitted)
Counsellor	£60.00	PSSRU 2011	Per consultation
Mental health worker	£76.00	PSSRU 2011	MH Nurse, £76 per 1 hour contact (assumed 1 hr)
Social Worker/Care Manager	£212.00	PSSRU 2011	Per 1 hour contact (assumed 1 hr)
Home help/Care Worker	£18.00	PSSRU 2011	Per weekday hour
Occupational Therapist	£82.00	PSSRU 2011	Community based OT, per 1 hour of client contact (assumed 1 hr)
Voluntary Group (e.g. MIND/CRUISE)	£21.73	PSSRU 2010	Cost per user session, voluntary/non-profit organisation (£21 session/2010).
Acute psychiatric ward (bed day)	£312.00	PSSRU 2011	Cost per bed day
Long-stay ward (bed day)	£222.52	PSSRU 2010	Cost per bed day (£215/2010)
Gen Med ward (bed day)	£321.00	PSSRU 2011	weighted ave. All adult mental health inpatient days
A & E (contact)	£106.00	PSSRU 2011	Contact, not admitted
Day hospital (Attend/day)	£126.00	PSSRU 2011	Cost per day, weighted ave of all adult attendances
Psychiatrist (outpatient contact)	£161.38	NHS Ref Costs**	2008–09 per consult (code MHOPFUA2, £155)
Psychologist (outpatient contact)	£135.00	PSSRU 2011	Cost per contact hour (assumed 1 hr)
Psychiatric Nurse/Care Coordinator (outpatient contact)	£76.00	PSSRU 2011	MH Nurse, £76 per 1 hour contact (assumed 1 hr)
Other Outpatient contact	£143.00	PSSRU 2011	Outpatient consultant services, weighted average
Day care centre (comm. serv/social care)	£34.00	PSSRU 2011	Cost per user session
Drop in Club (comm. serv/social care)	£34.00	PSSRU 2011	Assume cost as day centre, cost per user session
Help from friends/relatives (hrs)	£18.00	PSSRU 2011	Use cost per hour, based on unit cost for home help/care worker (as above).
Lost work (day) (friends/relatives)	£99.6	ONS 2011	Based on median gross weekly earnings in 2011 for f/t employees at £498.
Travel cost per mile (patient own car)	£0.44		Assumed reclaim/expense rate (running cost per mile)

*Unit costs at 2011 prices/costs.

**Costs uprated/adjusted to 2011 prices using HCHS index reported in PSSRU Unit Costs of Health Care [27].

PSSRU Unit Costs of Health Care 2011 [27].

NHS Ref Costs 2008–09 - http://www.ons.gov.uk/ons/dcp171778_256900.pdf (Accessed 18/05/12).

### Statistical methods

We analysed data on an intention-to-treat basis, in accordance with an analysis plan drawn up prior to the analysis of data [12]. We undertook analyses in STATA, version 12. Our primary economic analyses estimated mean cost and mean QALY by treatment allocation, and estimated differences between groups over 12-months, adjusting for baseline measures, and using pre-specified covariates for age (individual level), and at the cluster level covariates for deprivation (IMD), site, and practice size. We used a multi-level regression model (STATA, xtmixed) for the primary analyses, to consider the hierarchical (clustered) nature of the data, presenting the intraclass correlation coefficient (ICC) for the main analyses. We undertook data analyses using generalised linear models (GLM), with appropriate family and link components, to account for the potentially non-normally distributed nature of cost data. GLM model results were not different to those using the multi-level model applied in the main analyses.

We conducted a number of sensitivity analyses for areas of uncertainty in the cost effectiveness analyses: 1) we considered the effect of missing data, estimated by multiple imputation (STATA MI command, with 25 replicated datasets), using all available data on the target variable together with covariates for individual and cluster variables used in the base case regression analyses [28]; 2) we undertook analyses using a broader analytical perspective, including estimated costs for informal care and participant out-of-pocket expenses; 3) we analysed data for a scenario using the SF-6D [25] as an alternative QALY outcome measure; 4) we considered uncertainty in the intervention costs; 5) we analysed a scenario where one participant, with an extremely high level of self-reported resource use, was excluded, as this potentially offers a more likely and policy-relevant estimate of cost-effectiveness.

We combined estimates of incremental cost and incremental benefit to present incremental cost effectiveness ratios (ICERs), allowing decision-makers to assess value for money using the cost per QALY estimates (ICER = (Cost_CC_–Cost_TAU_)/(QALY_CC_–QALY_TAU_)). We used the NICE threshold of £20,000 to £30,000 per QALY [29], [30], i.e. the expected Payer willingness to pay per unit of additional outcome, to assess the cost-effectiveness of collaborative care, with ICERs below these values regarded as cost-effective. We used the non-parametric bootstrap approach [31], with 10,000 replications, to estimate 95% confidence intervals around estimated cost differences, and for QALY differences, in order to address uncertainty. To present the level of uncertainty on cost-effectiveness estimates we used the cost-effectiveness plane to present combinations of incremental cost and incremental QALY data from bootstrap replicates, and used the cost-effectiveness acceptability curve (CEAC), with the ‘net benefit statistic’ (net monetary benefit = (incremental QALY×willingness to pay per QALY) –incremental cost)) [32], [33], to present the probability that the intervention is cost-effective (i.e. incremental net benefit statistic is >0), against a range of potential cost-effectiveness thresholds.

## Results

We recruited a total of 581 participants to the CADET trial, from 49 practice clusters. The majority (55·6%) of participants fulfilled criteria for a moderately severe depressive episode with a further 29·9% meeting criteria for severe depression, 14.3% mild depression, and 72·6% having suffered from depression in the past. At baseline, 82% of participants had been prescribed antidepressant medication by their GP. The mean age was 44·8 years (SD 13·3), and 71·9% were women. A total of 276 participants were allocated to collaborative care and 305 allocated to usual care. Full trial results have been presented elsewhere [9]. In brief, however, we found that collaborative care improved depression immediately after treatment compared to usual care, with effects persisting to 12-month follow-up. After adjustment for baseline depression, the mean depression score was 1.33 PHQ-9 points lower (95% CI 0.35 to 2.31, p = 0.009) in collaborative care than in usual care at 4-months, and 1.36 lower (95% CI 0.07 to 2.64, p = 0.04) at 12 months. This difference equated to a standardised effect size of 0.26 (0.07 to 0.46). Patients receiving collaborative care had better outcomes than usual care in terms of depression recovery (odds ratio 1.67 (95% confidence interval 1.22 to 2.29); number needed to treat = 8.4) and response to treatment (odds ratio 1.77 (95% confidence interval 1.22 to 2.58); number needed to treat = 7.8).

### Cost for Collaborative Care

Our estimated mean cost per participant for the delivery of the collaborative care intervention was £272.50. This cost estimate comprises care manager costs at £232, and clinical supervision costs of £40.50. We based care manager costs on data available from electronic participant level records on 235 participants receiving collaborative care, from 10 Care managers, reporting mean (SD) care manager time per participant of 214.15 (115) minutes. We based clinical supervision costs on data collected from supervision records (n = 220 records) reporting a mean supervision time for care managers, in weekly sessions, at 35 minutes, with six participants discussed on average per session (based on data from 164 records). We assumed from this data that participants were discussed in three supervisory sessions (over the course of the intervention), at six minutes per participant per session.

Probabilistic analyses used to explore uncertainty around the main cost component, drawing from the distribution of contact time for care managers (mean 214.5 mins, SD 115 mins), showed that in 95% of simulations (cost estimates) the estimated cost of collaborative care was between £101 and £592 per participant (median £249).

### NHS and Social Care Resource Use and Costs

We found no statistically significant differences between treatment groups in use of resources during the six months prior to baseline assessment (see Table S1). Table 2 presents resource use over the 12-month follow-up, and Table 3 presents the costs associated with resource use over 12-month follow-up. Table 4 presents cost data by category with comparison by category by treatment group. We found a broadly similar pattern of resource use across groups, with estimated mean costs for NHS and social care (Third Party Payer perspective), excluding the collaborative care intervention, at £1,571 and £1,614 respectively for usual care and collaborative care participants. After adjustment for baseline cost and individual and cluster covariates the cost difference was not statistically significant, with wide confidence intervals. When including the cost for the collaborative care intervention, the mean total NHS and social care costs were £1,571 and £1,887 for usual care and collaborative care participants respectively, but similarly, after adjustment the cost difference of £271 was not statistically significant. Excluding the intervention cost, the one area of substantial cost difference between groups was on hospital stay, with a mean cost difference of £161 (regression adjusted estimate). This estimated difference in hospital cost was driven by one participant in the collaborative care group, who reported an acute psychiatric hospital stay of 100 days. When we excluded that one participant from analyses, the cost difference for hospital stay was adjusted to £34 (–119 to 189), and the related differences in NHS and social care, without collaborative care costs and with collaborative care costs, were adjusted to –£209 (less cost for collaborative care), and £63 (additional cost for collaborative care) respectively.

**Table 2:** Mean health and social care resource use (quantities) over 12 month follow-up.

Resource item	Usual Care		Collaborative Care	
	n = 305		n = 276	
	n	Mean (SD)[range]	n	Mean (SD)[range]
**Primary/Community Care:**				
GP (surgery/practice)	244	**8.21** (6.69)[0–56]	217	**7.77** (6.78)[0–45]
GP (at home)	247	**0.12** (0.80)[0–11]	218	**0.05** (0.27)[0–3]
Nurse (surgery/practice)	247	**1.77** (3.08)[0–24]	215	**1.68** (3.10)[0–32]
Nurse (at home)	247	**0.06** (0.46)[0–4]	218	**0.05** (0.45)[0–6]
Walk-in-centre (attendance)	247	**0.32** (0.87)[0–8]	217	**0.31** (0.86)[0–5]
Counsellor	246	**3.58** (11.26)[0–116]	212	**2.67** (7.21)[0–48]
Mental Health worker	247	**0.58** (3.51)[0–50]	215	**0.79** (3.72)[0–36]
Social worker	247	**0.34** (1.79)[0–14]	218	**0.58** (3.94)[0–33]
Home help/Care Worker	247	**4.35** (47.27)[0–722]	218	**1.24** (15.07)[0–220]
Occupational Therapist	247	**0.22** (0.98)[0–9]	218	**0.13** (0.61)[0–5]
Voluntary Group	247	**0.94** (5.80)[0–64]	218	**0.22** (1.39)[0–16]
**Secondary Care:**				
Hospital admissions, n+	247	34	218	28
Acute Psychiatric ward (days)	247	-	218	**0.46** (6.77)[0–100]^1^
Psychiatric rehab ward	247	-	218	-
Long stay ward	247	**0.06** (0.94)[0–15]	218	-
Psychiatric ICU ward	247	-	218	-
General Medical ward (days)	247	**0.48** (2.02)[0–21]	217	**0.42** (1.67)[0–12]
Other hospital ward/stay	247	**0.28** (1.58)[0–17]	218	**0.39** (2.12)[0–24]
A & E (attendance)	247	**0.40** (0.93)[0–7]	218	**0.34** (0.76)[0–5]
Day hospital (attendance)	247	**0.60** (2.22)[0–24]	218	**0.36** (1.19)[0–12]
Outpatient appointment	247	**2.62** (5.60)[0–58]	217	**2.63** (5.63)[0–65]
**Social care:**				
Used day care services (%)+	247	**3%/2%**	218	**4%/3%**
Day care centre	247	**0.28** (4.54)[0–70]	218	**0.07** (1.08)[0–16]
Drop in club	247	**0.56** (5.26)[0–70]	218	**0.12** (1.40)[0–20]
Day care other	247	**0.39** (2.85)[0–28]	217	**0.65** (5.67)[0–74]
**Informal care from friends/relatives:**				
Had help/care from friends/relatives (%)+		**45%/48%**		**38%/35%**
Hours per week help from friends/relatives#	230	**6.11** (15.44)[0–112]	209	**3.95** (10.11)[0–104]
Report time off work for friends/relatives (%)+		**7%/9%**		**7%/10%**
Days lost work by friends/relatives	246	**4.05** (29.14)[0–360]**	217	**1.65** (11.28)[0–144**]
**Patient other costs:**				
OTC medications (£)	246	**28.40** (57.90)[0–429]**	215	**40.31** (68.61)[0–507]**
Travel costs (£)	246	**10.98** (30.30)[0–320]	216	**14.33** (37.57)[0–202]
Own care travel (miles)	246	**26.12** (77.68)[0–600]	214	**31.53** (138.95)[0–1862]
Other ‘one-off’ costs (£)	246	**35.77** (144.35)[0–1569]	218	**51.58** (213.53)[0–1,998]

1 includes one participant with 100 days in psychiatric ward/admission.

+ Here this data refers to proportion with hospital stay [resource use] at 4 mths follow-up and at 12 months follow-up (mths 5–12) questionnaire.

# this is a weekly number of hours (weighted average of data reported at 4-mth and 12 mth follow-up), and requires×52 weeks for annual estimate of hours.

**Kruskal-Wallis (non-parametric) test: no statistically significant differences between groups, other than for OTC cost, and cost for days lost work by friends/relatives, which are statistically significant at p≤0.05.

**Table 3:** Estimated mean cost (£’s) for health, social care, and other resource use, over 12 month follow-up.

Resource item	12-mth Follow-up			
	Usual Care		Collaborative Care	
	n	£ Mean (SD)	n	£ Mean (SD)
**Primary/Community:**				
GP (surgery/practice)	244	295.52 (241)	217	279.76 (243)
GP (at home)	247	14.21 (97)	218	5.55 (32)
Nurse (surgery/practice)	247	26.54 (46)	215	25.19 (46)
Nurse (at home)	247	1.94 (14)	218	1.38 (13)
Walk-in-centre (attendance)	247	13.28 (35)	217	12.66 (35)
Counsellor	246	214.63 (676)	212	160.47 (433)
Mental Health worker	247	44 (267)	215	59.74 (283)
Social worker	247	72.10 (379)	218	122.53 (835)
Home help/Care Worker	247	78.27 (851)	218	22.38 (271)
Occupational Therapist	247	18.26 (80)	218	10.53 (50)
Voluntary Group	247	20.50 (126)	218	4.88 (30)
**Secondary Care:**				
Acute Psychiatric ward	247	0	218	143.12 (2,113)
Psychiatric rehab ward	247	0	218	0
Long stay ward	247	13.51 (212)	218	0
Psychiatric ICU ward	247	0	218	0
General Med ward	247	154.65 (649)	217	134.61 (535)
Other hosp ward/stay	247	90.97 (507)	218	123.69 (682)
A & E	247	43.06 (99)	218	36.47 (81)
Day hospital	247	74.99 (280)	218	45.08 (150)
Out Patient Appt, Psychiatrist	247	26.79 (148)	217	43.88 (170)
Out Patient Appt, Psychologist	247	25.14 (313)	217	25.51 (296)
Out Patient Appt, Psychiatric Nurse	247	8.92 (67)	217	12.61 (166)
Out Patient Appt, Other	246	306.93 (588)	215	285.34 (498)
**Social care:**				
Day care centre	247	9.64 (151)	218	2.50 (37)
Drop in club	247	19.13 (179)	218	4.06 (48)
Day care other	247	13.35 (97)	217	22.09 (193)
**Informal care from friends/relatives:**				
help from friends/relatives	230	5,714.73 (14,455)	209	3,698.50 (9,462)
Days lost work by friends/relatives	246	403.26 (2,902)	217	164.78 (1,123)
**Patient other costs:**				
OTC medications	246	28.40 (58)	215	40.31 (69)
Travel costs	246	10.98 (30)	216	14.33 (38)
Own car travel	246	11.75 (35)	214	14.19 (63)
‘one-off’ costs	246	35.77 (144)	218	51.58 (213)

***Kruskal-Wallis (non-parametric) test: no statistically significant differences between groups, other than for OTC cost, and cost for days lost work by friends/relatives, which are statistically significant at p≤0.05.***

***(Appt*** = ***appointment; OTC*** = ***over the counter).***

**Table 4:** Estimated costs, and cost differences (adjusted, unadjusted), over 12-month follow-up, by group.

Resource item	Usual Caren = 305		CollaborativeCare n = 276		Difference(no adjustment)	Difference, adjusted for baseline andparticipant/cluster covariates*
	n	Mean (SD) £	n	Mean (SD) £	£	Mean (95% CI)** £
Primary and communityservices/care	243	801.49 (1,476.98)	208	715.86 (1,220.06)	–85.63	–116.48 (–341.06, 110.91)
Secondary care: hospitalstay	247	259.14 (835.40)	217	402.65 (2,282.88)	143.51	160.92 (–70.81, 481.70)
Secondary care:outpatient care	246	368.03 (781.43)	215	368.09 (692.60)	0.06	–30.68 (–148.85, 111.70)
Secondary care: dayhospital	247	74.99 (280.31)	218	45.08 (149.65)	–29.91	–14.52 (–50–13, 17.94)
A & E	247	42.06 (98.66)	218	36.47 (80.51)	–5.59	–5.87 (–22.39, 9.99)
Day services and care	247	42.12 (334.85)	217	28.67 (203.43)	–13.45	1.83 (–38.51, 41.01)
*Total NHS and personal* *social services (excl* *Collab Care)*	242	1,570.70 (2,441.55)	205	1,614.32 (3,714.49)	43.62	1.78 (–454.82, 640.81)
CADET collaborativecare		**-**		272.50	272.50	-
**Total NHS and personal** **social services**	242	**1,570.70** (2,441.55)	205	**1,886.82** (3,714.49)	**316.12**	**270.72 (–202.98, 886.04)** #1
Patient personal costs(OTC/meds, travel costs,plus patient ‘one-off’ costs)	244	86.64 (175.50)	211	120.79 (260.37)	34.15	24.95 (–12.41, 65.61)
Informal care costs	230	5,714.73 (14,455.18)	209	3,698.50 (9,642.61)	–2,016.23	–1,114.13 (–3,366.09, 1,117.32)
**TOTAL costs (NHS** **and patient/related costs)**	223	**7,010.59** (13,492.42)	195	**5,764.48** (10,796.40)	**–1,246.11**	**–312.83 (–2,339.92, 2,035.27)** #2

*regression analyses using multilevel model (‘xtmixed’ STATA), with baseline value as covariate, age, and cluster level covariates.

**95% Confidence intervals estimated using non-parametric bootstrap method.

#1 ICC = 0.0000 (95% CI 0.0000, 0.0000).

#2 ICC = 0.0000 (95% CI 0.0000, 0.0000).

*On cost estimates for individual items/categories, ANOVA showed no statistically significant differences between groups; [note: informal care cost difference at p = 0.088].*

### Broader Participant Level and Social Costs

Tables 2 and 3 report resource use and cost estimates associated with informal care from friends and/or relatives, and other patient out-of-pocket expenses. Our findings show that informal care costs, when estimated using a shadow price for informal care (an estimate of £18 per hour, see Table 1), represented the largest resource and cost burden associated with participants’ depression. Participants in the treatment as usual group reported a high use of informal care, which resulted in a higher mean (SD) cost estimate, over 12-months, at £5,715 (£14,455) compared to £3,699 (£9,462) in the collaborative care group. However there is wide variation in the self-report data as shown by the large standard deviations. When adjusting for baseline costs and other covariates the difference in estimated cost for informal care was –£1,114 (95% CI –£3,366 to £1,117), with lower costs for the collaborative care group, and, therefore, lower total costs in the collaborative care group (Table 4).

### Quality-Adjusted Life-Years

Table 5 reports data on health state values for the EQ-5D and the SF-6D, and the estimated QALY values over 12-months. When adjusted for baseline and for individual and cluster covariates we found a difference of 0.02 (95% CI –0.02 to 0.06) in the QALY over 12 months for the EQ-5D, and 0.017 (95% CI 0.000 to 0.032) for the SF-6D. Both measures showed a QALY gain for collaborative care, although the EQ-5D difference is not statistically significant.

**Table 5:** Health State Values and QALY comparisons (adjusted, unadjusted), over 12-month follow-up, by group.

Measure (time-point)	Usual Care n = 305		Collaborative Care n = 276		Difference(no adjustment)	Difference, adjusted forbaseline and participant/cluster covariates*
	n	Mean (SD) [range]	n	Mean (SD)[range]		Mean (95% CI)**
EQ-5D: Baseline	305	0.464 (0.313)[–0.29, 1.00]	276	0.504 (0.288)[–0.349, 1.00]	0.040	
EQ-5D: 4-month	273	0.557 (0.331)[–0.239, 1.00]	228	0.599 (0.341)[–0.484, 1.00]	0.042	
EQ-5D: 12-month	254	0.593 (0.338)[–0.349, 1.00])	227	0.650 (0.317)[–0.484, 1.00])	0.057	
**EQ-5D: QALY** **(12-month)**	**248**	**0.554 (0.286)** **[**–**0.27, 0.97]**	**218**	**0.605 (0.261)** **[**–**0.29, 0.97]**	0.051*	**0.019 (**–**0.019, 0.06)** #
SF-6D: Baseline	303	0.538 (0.86)[0.30, 0.77]	274	0.540 (0.83)[0.30, 0.82]	0.002	
SF-6D: 4-month	269	0.597 (0.126)[0.30, 1.00]	227	0.614 (0.140)[0.32, 1.00]	0.017	
SF-6D: 12-month	250	0.605 (0.131)[0.30, 1.00])	223	0.634 (0.144)[0.30, 1.00])	0.029*	
**SF-6D: QALY** **(12-month)**	241	**0.591 (0.109)** **[0.30, 0.90]**	211	**0.609 (0.114)** **[0.35, 0.91]**	0.018	**0.0168 (0.000, 0.032)**

# ICC = 0.0000 (95% CI 0.0000, 0.0000).

*ANOVA, p≤0.05.

**regression analyses using multilevel model (‘xtmixed’ STATA), with baseline.

### Cost-Effectiveness Analyses

**Figure 1:**
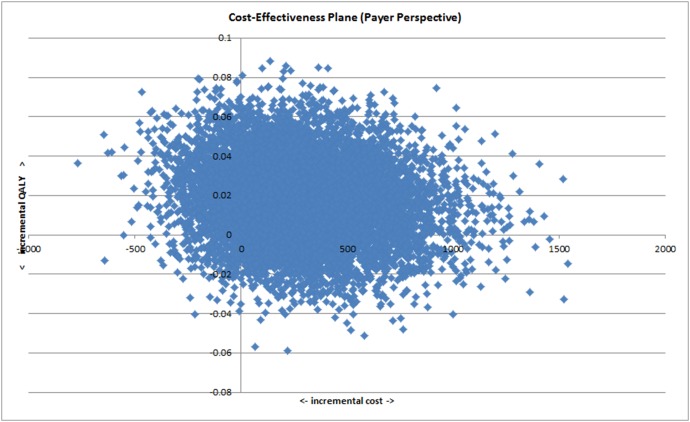
Cost-effectiveness plane (Payer perspective).

**Figure 2:**
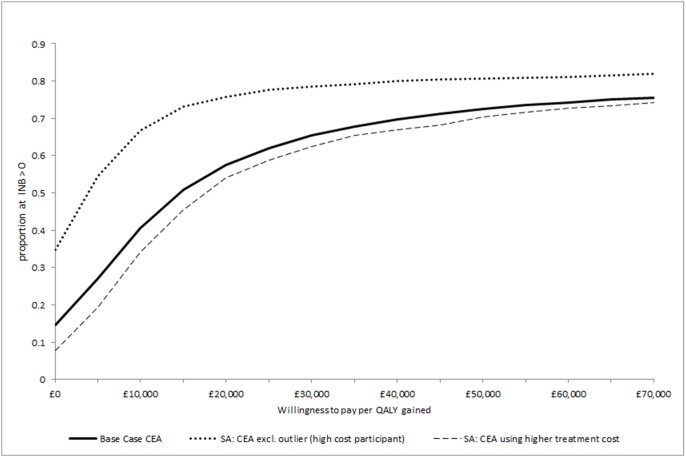
Cost-effectiveness acceptability curves.

Table 6 presents data used to estimate cost per QALY, and the cost per QALY estimates, based on participants with data on costs and outcomes at follow-up. The base case cost per QALY for collaborative care was £14,248, adopting a NHS and social care perspective, with uncertainty around this estimated illustrated in Figure 1 (cost-effectiveness plane), and Figure 2 (CEAC). The probability that collaborative care is cost effective, compared to treatment as usual, is 0.58 at a willingness to pay of £20,000 per QALY, and 0.65 at a willingness to pay of £30,000 per QALY.

**Table 6:** Cost Effectiveness Analyses.

Scenario/Analyses	Difference, adjusted forbaseline andparticipant/clustercovariates* Mean(95% CI)	ICER, Cost (£)per QALY	Probability Cost-effective at WTP‡per QALY gained:	
			£20,000 per QALY	£30,000 per QALY
**Base Case**:				
Total NHS and personal social services	£270.72(–202.98, 886.04)			
EQ-5D: QALY (12-month)	0.019(–0.019, 0.06)	**£14,248**	0.58	0.65
Sensitivity Analyses:				
(1) Base Case CEA, withmultiple imputation ofmissing data				
Total NHS and personal social services	£292.08(–216.88, 801.04)			
EQ-5D: QALY (12-month)	0.017(–0.020, 0.054)	£17.490	N/A	N/A
(2) CEA Using SF6D QALY data				
SF6D: QALY (12-month)	0.0168(0.000, 0.032)	£16,114	0.57	0.72
(3) CEA when excludingone high cost participant				
Total NHS and personal social services	£63.34(–295.98, 422.67)			
EQ-5D: QALY (12-month)	0.019(–0.018, 0.06)	£3,334	0.76	0.79
(4) CEA using higher costestimate for CollaborativeCare, at meancost of £338.80				
Total NHS and personal social services	£337.02(–136.67, 952.34)	£17,738	0.54	0.62
(5) CEA using a broaderperspective, including patientcosts and informalcare costs	–£312.83(–2,339.93, 2,035.27)	Collaborative Care is dominant**	N/A	N/A

*Adjusting for baseline measures, and pre-specified covariates for age (individual level), and (at the cluster level) deprivation (IMD), site, and practice size.

**Dominance: lower expected costs, with greater expected QALY gain.

‡ WTP = willingness to pay; based in the assessment of incremental net benefit statistic, and WTP thresholds commonly applied in the UK NHS (NICE, 2013).

### Sensitivity Analyses

Table 6 presents the results of our sensitivity analyses, where we estimated incremental costs and QALYs, and cost per QALY using alternative assumptions. In base case analyses 23% of cost data are missing at 12-month follow-up (21% in control; 25% in collaborative care), and 20% of QALY (EQ-5D) data are missing at the 12-month follow-up (19% in control; 21% missing in collaborative care). Imputation of missing data resulted in an estimated incremental cost of £292 and an incremental EQ-5D QALY gain of 0.017, with a cost per QALY of £17,490. Where we adopted a broader analytical perspective, including all participants with data on cost and outcome at follow-up, we estimated a mean cost saving of £313 with collaborative care, alongside the estimated incremental gain in QALYs (0.02). This, therefore, represents a position of dominance for the collaborative care intervention compared to treatment as usual. Using the SF-6D QALY estimate the cost per QALY increased to £16,114, with a 0.57 and 0.72 probability of being cost-effective at a willingness to pay of £20,000 and £30,000 per QALY respectively. When we analysed CEA using an alternative cost for the collaborative care intervention, assuming a cost of £338.80 per participant (compared to base case of £272.50) to allow for additional clinical supervision time to the care manager, and therefore per participant, and for supervision from a psychiatrist (unit cost per hour, £267, [27]) the base case cost per QALY estimate increased to £17,738. When we undertook a scenario the same as the base case but with one participant, with extremely high resource use, excluded from analyses the cost per QALY was reduced to an estimate of £3,334 with a 0.76 and 0.79 probability of being cost-effective at a willingness to pay of £20,000 and £30,000 per QALY respectively.

## Discussion

Our results show that collaborative care is cost-effective, compared to usual care, in treating people with depression in a UK primary care setting, where providers are willing to pay up to £20,000 per QALY gained. When taking a broader analytical perspective, and including costs associated with informal care, results show that collaborative care is expected to be cost saving, with expected health gains, and therefore dominates the usual care comparator. Previous reviews [10], [34] have reported on economic analyses related to collaborative care, across a range of settings, including care for depression. These reviews have identified evidence from cost-utility (cost per QALY) studies to support the economic value of collaborative care for depression in the United States healthcare system, and have also highlighted an absence of evidence for the UK. Results presented here for the CADET trial represent the first study to estimate the cost-effectiveness of collaborative care in a UK primary care setting.

Our cost-effectiveness analyses report an expected modest mean QALY gain at a relatively low cost. The relative effectiveness of collaborative care versus usual care reported in the CADET trial [9] is regarded as clinically meaningful in terms of recovery (defined as a follow up score of less than or equal to 9 on PHQ-9) and treatment response (defined as a 50% reduction or greater at follow up compared to baseline PHQ-9). Although, as seen in the QALY data, the average individual treatment response was modest, at 12 months 56% of patients receiving collaborative care were ‘recovered’, 15% more than in usual care, and 49% responded to treatment, 13% more than in usual care. Whilst the mean QALY gains are modest, they are comparable, and favourable, to those recently reported for evaluation of a UK Improving Access to Psychological Therapies (IAPT) service [35], which estimated a mean EQ-5D QALY gain of 0.014 (SF6D gain of 0.008). In the IAPT evaluation, the SF6D values at baseline and follow-up are a little higher than those for CADET, probably reflecting the fact that the patient group had a lower mean PHQ-9 score compared to CADET participants. Our estimated costs for health and social care in CADET are similar to those reported in the IAPT service evaluation, for IAPT service or comparator mental healthcare services [35]. QALY gains from the collaborative care intervention are in a similar range to those reported for evaluation of therapist-delivered CBT for depression [36], where a mean (95% CI) incremental QALY benefit is reported at 0.027 (–0.012 to 0.066).

Our base case difference in health and social care (NHS and PSS) costs, over 12 months (£271), and the subsequent cost per QALY estimate, of £14,248 are heavily influenced by one participant who reported extremely high levels of service use for specialist care, including a 100-day stay in an acute psychiatric hospital. This participant, in the collaborative care group, had an estimated service use cost of £48,522, compared to a mean cost of £1,637 for all other trial participants with cost data over 12-months (n = 446); 94% of participants have cost estimates at less than £5,000; all but four participants have costs at less than £10,000; three participants have costs estimated between £10,000 and £24,000. When we excluded this one participant from analyses, the difference in NHS and PSS costs, when including cost for collaborative care, was £63, between collaborative care and usual care, with an estimated cost per QALY of £3,334. For our primary analysis we retained the intent to treat principle, and this one participant is included in the base case analyses, but we would suggest that the likely cost-effectiveness of collaborative care in practice might be closer to the estimate in the sensitivity analyses with this one participant excluded.

Although there is uncertainty in the cost and QALY data, our probabilistic analyses indicate that collaborative care has a 58% or 65% probability of being cost-effective, at commonly assumed UK NHS willingness to pay of £20,000 or £30,000 per QALY. Where we considered uncertainty around the CEA estimate that excludes one participant with high service use and costs (£3,334 per QALY), the probability of being cost effective at these cost per QALY thresholds is above 75%. Given the wide variation in the costs reported for general health and social care resource use, and the uncertainty in the profile of resource use, a simplistic view would be to have an expectation that the introduction of collaborative care will involve an additional cost of £272.50 per participant for the UK NHS, and this potential cost alongside estimated EQ-5D QALY gains, results in an expected cost per QALY gain of £14,342, which is similar to the base case analyses presented here and represents a cost effective use of NHS resources.

## Strengths and Limitations

The CADET trial was a high quality RCT with an integrated economic evaluation, reported here. The analyses follow good practice for economic evaluation, and our within-trial analysis demonstrates cost-effectiveness at the willingness to pay threshold of £20,000 per QALY, without the need to extrapolate potential benefits over the longer term. Our analyses use data collected within trial to estimate resource use and cost associated with delivery of the intervention, but rely on self-report data, from interviewer administered questionnaires, to estimate health and social care service use, and to estimate broader resource impacts. There is no consensus in the economic evaluation literature on the relative merits of different methods for collection of resource use data [37]–[39], but routinely collected service use data may have provided a more rigorous estimate of service use, particularly for primary care contacts [40]. However, there are difficulties and costs related to collection of service use data from 42 general practices, and from other necessary routine data collection for aspects of care not recorded in GP records, therefore we chose to use participant self-report data. Difficulties collecting detailed data via self-report on medications, related to potential for variation in medication names, dose, the potential for people to be on medications for a wide range of co-morbid conditions, and other factors, can lead to errors in self-reporting. These issues led to the exclusion of medication costs from the economic analysis plan [12], and this may be a limitation in the results presented here, as medication adherence has been shown to be one of the potential benefits from collaborative care [8]. However, most participants in both collaborative care and the usual care groups remained on antidepressant prescriptions (74.8% v 73.8% at four months; 69.7% v 69.2% at 12 months).

The perspective on the analyses do not extend to the broad welfare and economic impacts of depression, including impact on productivity costs, as such costs are not included in the reference case analyses suggested by NICE [29] for UK analyses. However, data collection did cover aspects of care and support, and patient costs that have extended the primary perspective (of NHS and PSS costs) to a broader patient and societal orientated perspective. We accept that the use of a relatively small number of categories for these broader considerations may be a limitation in the analyses. However, as in other studies (e.g. [41]) we found that resources, and estimated costs, associated with informal care were a dominant aspect of care and costs, when taking a wider perspective, and provided clear guidance on the magnitude of costs at a wider perspective.

As discussed above, our estimated differences in costs, and in EQ-5D QALYs, did not reach statistical significance, introducing some uncertainty. However, the methods used in cost-effectiveness analyses account for uncertainty through replication of the estimates of incremental costs and QALYs, using the non-parametric bootstrap approach. The presentation of uncertainty using the CEAC, allows decision makers to consider the likelihood of collaborative care providing a cost-effective use of resources.

## Implications

The CADET trial has shown that collaborative care improves depression immediately after treatment compared to usual care, it also has effects that persist to 12 month follow-up and is preferred by patients over usual care, and here our economic analysis of collaborative care also shows that it is cost effective in the UK. We have therefore, responded to the evidence needs, and answered the specific request for evidence required by NICE [11], on the clinical and cost effectiveness of collaborative care, in that our positive findings on the cost-effectiveness of collaborative care adds to the evidence for its sustained clinical effectiveness.

Given that collaborative care is more effective over a sustained period of time than usual care and represents value for money against commonly used thresholds for cost per QALY, we suggest that commissioners of healthcare in the UK and elsewhere organise their depression management services using a collaborative care model. Evidence here indicates that services should be commissioned to support the management of patients with depression in UK primary care using a collaborative care model, as it would be both effective and cost effective to do so. Although the introduction of collaborative care will involve additional resources for the delivery of the intervention, there is reasonable potential for cost saving against other areas of health and social care over time.

Where we have included informal care costs in our analyses, the finding that collaborative care is the dominant intervention, compared to care as usual, is an important issue. Family members and others involved in informal care are contributing to the care of depression in a substantial manner. As Richard Layard and others have often asserted [42], the implications of these care costs on productivity is a significant burden on the economic activity of a nation. The introduction of collaborative care has the potential to relieve some of the significant burden that falls on informal carers, and can reduce this economic load.

## Supporting Information

Table S1 **Estimated mean baseline cost (£’s) for health, social care, and other resource use.**
(DOCX)
