# Supplementary material for: Cost-Effectiveness of Collaborative Care for Depression in UK Primary Care: Economic Evaluation of a Randomised Controlled Trial (CADET)
Source: PLoS One. 2014 Aug 14;9(8):e104225. doi: 10.1371/journal.pone.0104225 (PMC4133193; doi:10.1371/journal.pone.0104225)
Supplement: Table S1 — Estimated mean baseline cost (£’s) for health, social care, and other resource use. (DOCX) [file pone.0104225.s001.docx]

**Table S1. Estimated mean baseline cost (£’s) for health, social care, and other resource use**

| **Resource item** | **Baseline (6-mths prior to baseline assessment)** | | | |
| --- | --- | --- | --- | --- |
|  | **Control** | | **Intervention** | |
|  | **n** | **Mean (SD) £** | **n** | **Mean (SD) £** |
| **Primary/Community** |  |  |  |  |
| GP (in surgery/practice) | 303 | 206.85 (157) | 273 | 197.41 (162) |
| GP (home visit) | 305 | 6.74 (45) | 276 | 10.96 (81) |
| Nurse (in surgery/practice) | 305 | 16.08 (33) | 275 | 15.98 (30) |
| Nurse (home visit) | 305 | 2.46 (20) | 276 | 20.00 (325) |
| Walk-in-centre (attendance) | 305 | 9.41 (41) | 272 | 7.39 (24) |
| Counsellor | 304 | 63.55 (196) | 274 | 81.02 (263) |
| Mental Health worker | 305 | 14.20 (74) | 275 | 26.56 (149) |
| Social worker | 305 | 47.96 (394) | 275 | 69.38 (798) |
| Home-help / Care Worker | 305 | 15.11 (191) | 275 | 26.18 (279) |
| Occupational Therapist | 305 | 6.45 (55) | 275 | 8.05 (43) |
| Voluntary Group | 305 | 6.34 (57) | 271 | 10.02 (65) |
| **Secondary Care:** |  |  |  |  |
| Acute Psychiatric ward | 305 | 4.09 (71) | 276 | 19.22 (229) |
| Psychiatric rehab ward | 305 | 0 | 276 | 0 |
| Long stay ward | 305 | 0 | 276 | 0 |
| Psychiatric ICU ward | 305 | 0 | 276 | 0 |
| General Med ward | 304 | 143.61 (671) | 275 | 89.88 (411) |
| Other hospital ward/stay | 305 | 110.51 (1,069) | 276 | 56.99 (403) |
| Accident & Emergency | 305 | 30.58 (92) | 276 | 26.12 (72) |
| Day hospital | 305 | 41.72 (188) | 276 | 41.72 (188) |
| OP-Psychiatrist | 305 | 9.52 (99) | 276 | 22.22 (207) |
| OP-Psychologist | 305 | 3.98 (46) | 276 | 3.91 (41) |
| OP-Psychiatric Nurse | 305 | 0.50 (6) | 276 | 7.99 (103) |
| OP-Other | 305 | 160.35 (417) | 276 | 170.46 (335) |
| **Social care:** |  |  |  |  |
| Day care centre | 305 | 0.22 (4) | 276 | 0.74 (12) |
| Drop in club | 305 | 5.69 (60) | 276 | 0 |
| Day care other | 305 | 11.82 (119) | 276 | 2.59 (41) |
| **Informal care from friends/relatives:** |  |  |  |  |
| help from friends/relatives | 298 | 3,392.21 (9,888) | 269 | 3,016.77 (8,469) |
| Days lost work by friends/relatives | 303 | 226.48 (1,635) | 275 | 145.96 (1,121) |
| **Patient other costs:** |  |  |  |  |
| OTC medications (£) | 299 | 18.24 (38) | 273 | 20.58 (40) |
| Travel costs (£) | 296 | 7.62 (32) | 267 | 8.52 (39) |
| Own car travel | 296 | 6.02 (23) | 267 | 8.25 (31) |
| ‘one-off’ costs (£) | 305 | 16.36 (109) | 276 | 109.54 (1,353) |

***No statistically significant differences between groups (non-parametric, Kruskal Wallis test);***

***OP = outpatient; OTC = over the counter [medications];***
